# Pediatric Hodgkin lymphoma– biomarkers, drugs, and clinical trials for translational science and medicine

**DOI:** 10.18632/oncotarget.11509

**Published:** 2016-08-22

**Authors:** Poonam Nagpal, Mohamed R. Akl, Nehad M. Ayoub, Tatsunari Tomiyama, Tasheka Cousins, Betty Tai, Nicole Carroll, Themba Nyrenda, Pritish Bhattacharyya, Michael B. Harris, Andre Goy, Andrew Pecora, K. Stephen Suh

**Affiliations:** ^1^ The Genomics and Biomarkers Program, The John Theurer Cancer Center, Hackensack University Medical Center, Hackensack, NJ, USA; ^2^ Department of Clinical Pharmacy, Jordan University of Science and Technology, Irbid, Jordan; ^3^ Department of Research, Hackensack University Medical Center, Hackensack, NJ, USA; ^4^ Department of Pathology, Hackensack University Medical Center, Hackensack, NJ, USA; ^5^ Department of Pediatrics, Hackensack University Medical Center, Hackensack, NJ, USA; ^6^ Clinical Divisions, The John Theurer Cancer Center, Hackensack University Medical Center, Hackensack, NJ, USA

**Keywords:** Hodgkin lymphoma, pediatric, adolescent, biomarker, tumor microenvironment

## Abstract

Hodgkin lymphoma (HL) is a lymphoid malignancy that is typically derived from germinal-center B cells. EBV infection, mutations in NF-κB pathway genes, and genetic susceptibility are known risk factors for developing HL. CD30 and NF-κB have been identified as potential biomarkers in pediatric HL patients, and these molecules may represent therapeutic targets. Although current risk adapted and response based treatment approaches yield overall survival rates of >95%, treatment of relapse or refractory patients remains challenging. Targeted HL therapy with the antibody-drug conjugate Brentuximab vedotin (Bv) has proven to be superior to conventional salvage chemotherapy and clinical trials are being conducted to incorporate Bv into frontline therapy that substitutes Bv for alkylating agents to minimize secondary malignancies. The appearance of secondary malignancies has been a concern in pediatric HL, as these patients are at highest risk among all childhood cancer survivors. The risk of developing secondary leukemia following childhood HL treatment is 10.4 to 174.8 times greater than the risk in the general pediatric population and the prognosis is significantly poorer than the other hematological malignancies with a mortality rate of nearly 100%. Therefore, identifying clinically valuable biomarkers is of utmost importance to stratify and select patients who may or may not need intensive regimens to maintain optimal balance between maximal survival rates and averting late effects. Here we discuss epidemiology, risk factors, staging, molecular and genetic prognostic biomarkers, treatment for low and high-risk patients, and the late occurrence of secondary malignancies in pediatric HL.

## INTRODUCTION

Hodgkin lymphoma (HL) is a unique monoclonal lymphoid malignancy that is characterized by the presence of typical bi/multinucleated Reed-Sternberg cells and their variants, mononucleated Hodgkin cells, collectively known as Hodgkin Reed-Sternberg cells (HRS). Most HRS cells express CD15 and CD30 (85% and 100%, respectively in HL cases). HRS cells have a B-cell ancestry as revealed by single cell PCR detection of Ig gene rearrangements [1]; and somatic mutations in the variable region of heavy and light chain Ig genes are suggestive of a late germinal or post-germinal center B-cell origin [2–4]. Despite their B-cell origin, HRS cells typically lack core B cell features, including specific signaling molecules that are associated with the B-cell lineage, which are either absent or expressed by only a small subset of cells [5–7]. In addition to the variable B-cell marker expression shown by ∼95% of classical Hodgkin lymphoma (cHL) cases, HRS cells can express markers that are characteristic of other hematopoietic lineages, including dendritic cells, monocytes, and T-cells (5-15% cHL cases) [8–10]. Though rarely expressed, T-cell markers on HRS cells have been reported to be independently associated with poor prognosis [10, 11].

Biologically, HL is similar if not identical in children and adults except for the relative incidence of specific histological subtypes and distinct immune response against HRS cells in tumor microenvironment. HL can be divided into two broad classes based on histologic features and phenotypes: (1) Classical Hodgkin lymphoma (cHL) and (2) Nodular lymphocyte predominant Hodgkin lymphoma (NLPHL). cHL can be further divided into four subtypes: lymphocyte rich (LR), nodular sclerosis (NS), mixed cellularity (MC), and lymphocyte depleted (LD). NLPHL is considered to be a distinct disease entity that is more similar to B-cell non-HL than to cHL [12]. Therefore, this review will focus on cHL.

With the advent of recent advanced treatment and imaging technologies, a cure rate of >95% has been achieved for pediatric HL patients. However, the prognosis of relapse/refractory (RR) patients is dismal The secondary malignancy and cardiopulmonary toxicity that can be caused by HL treatment motivated greater focus on therapy refinements that would minimize toxicity while maintaining high cure rates. As such, identification of prognostic biomarkers in pediatric patients that correlate with clinical outcome will increase the understanding of HL pathology and likely influence therapeutic approaches. In this review, we discuss the main characteristics and prognostic biomarkers in pediatric cHL as well as current treatments and emerging targeted therapies. We also briefly present treatment complications and secondary malignant neoplasms (SMNs) that can occur in long-term survivors of HL.

## EPIDEMIOLOGY AND RISK FACTORS

Childhood HL represents 6% of all cancers and has an incidence rate of 12 cases/million/year in the 0-14 year age group with a typical male predominance [13]. Among the demographics of different age groups, HL shows a characteristic bimodal distribution, with the first, larger peak seen for adolescents and young adults (15-24 year age group) and a second, smaller peak occurring for adults (around 59 years) (Table 1) [14, 15]. The MC histological subtype, mostly associated with Epstein-Barr virus (EBV), is observed primarily in young children and represents about 20% of HL; whereas, the NS subtype is seen predominantly in adolescents and young adults and represents about 75% of HL [16]. Although EBV positivity is not a direct finding of HL, tumor cells are infected with EBV in approximately 30% and 90% of all HL cases in developed and developing countries, respectively [16, 17]. Two key EBV genes involved in the etiology of HL are: *latent membrane protein* (LMP) *1* (*lmp1*), which induces constitutive nuclear factor-kappaB (NF-κB) activation by mimicking CD40 receptor [18], and *lmp2A,* which can take over the function of the B-cell receptor (BCR) [19]. Various findings revealed raised antibody titers and EBV DNA detection in HL patients that are suggestive of association of EBV with HL [20, 21]. Additionally, the increased deregulation of the NF-κB pathway in EBV+ patients relative to EBV- patients is consistent with a role for EBV in the development of HL [22]. In EBV- cases signaling events are complemented by mutations in tumor necrosis factor alpha-induced protein 3 (TNFAIP3), which encodes the NF-κB inhibitor A20 [23]. Despite these findings, the prognostic significance of EBV positivity is puzzling, and is less well investigated in pediatric HL patients. Only a few studies have reported a direct prognostic significance of EBV positivity in HL [24–26], whereas numerous studies reported either no association or better clinical outcome of EBV+ pediatric HL [27–29]. The different outcomes might be attributed to variations in the presence of EBV+ HL that is related to geography, age, ethnicity, and histological type.

**Table 1:** Comparison of demographic and clinical characteristics between different age groups of Hodgkin lymphoma (HL) patients

Characteristics [Ref]	Pediatric HL	AYA (Adolescent and young adult) HL	Adult HL
Age	0-14	15-34	>35
Prevalence [13]	6/million/yr	32/million/yr	28/million/yr
Predominant gender [13, 149]	Male	Female (15-19), Male (<20)	Male (common: 55-74 yr)
Histology [16]	Mixed-cellularity Hodgkin lymphoma (22%)	Nodular sclerosis Hodgkin lymphoma (76%)	Nodular sclerosis Hodgkin lymphoma (61%)
Predominant stage at Diagnosis [16]	II	II	II
Most common symptoms [16, 150]	Painless adenopathy involving supraclavicular or cervical area (80%)	Asymptomatic mediastinal disease	Asymptomatic lymphadenopathy (<80%), B symptoms (40%), Intermittent fever (35%)
EBV association [151]	Yes (<10yr: 80%)	No (<30%)	No (20-50 yr), Yes (>60yr; 70%)
Treatment outcome (5 yr OS^a^) [16, 149]	90-95%	90%	85.90%

a OS, Overall Survival

Epidemiologic studies linked the risk of childhood HL to limited social contact, fewer siblings, low housing density, immunodeficiency, viral infection, high fetal growth rate, and familial HL (4.5% of cases) [30–32]. Genetic susceptibility to HL is strongly supported by a twin study of lymphomas, wherein, monozygotic twins of HL patients had nearly a 100-fold increased risk of HL, but dizygotic twins had no excess risk suggestive of co-inheritance of genetic variants [33]. The co-inheritance of genetic variants and shared childhood environmental effects could confer HL susceptibility was further proven by the 3-fold elevated risk among first-degree relatives of HL patients [32]. The highest familial risk (81-fold) was found in first-degree relatives with a concordant LR subtype [32]. Other genetic risk factors that are associated with familial predisposition to HL, including single nucleotide polymorphisms, are reviewed elsewhere [34].

## CURRENT STAGING

The Ann Arbor staging system with Cotswolds' modification is the current standard for staging HL in children. This system includes four stages wherein Stages I and II indicate limited disease while stages III and IV as advanced disease. Stage I constitutes 19%, stage II 49%, stage III 19%, and stage IV 13% of all pediatric HL cases [16]. HL patients are further stratified into 3 risk groups based on disease stage and extent, disease bulk, and systemic B symptoms (e.g., unexplained persistent fevers, weight loss, or drenching night sweats). According to the Children's Oncology Group (COG) classification, the low-risk group includes patients with stage IA/IIA without bulk or extranodal extension (E) while patients at intermediate-risk are included in a broad group that encompasses stage IA bulk or E, IB, IIA bulk or E, IIB, IIIA and IVA. High-risk includes all stage IIIB and IVB patients.

Staging and monitoring of pediatric HL patients is often done using fluorodeoxyglucose (FDG) positron emission tomography (PET)-computed tomography, which integrates functional and anatomic tumor characteristics to help delineate radiotherapy (RT) margins and provide a baseline scan for subsequent response assessment. The primary goals of FDG-PET for interim assessment after initial cycles of chemotherapy are to further refine risk classification and to identify patients who are either cured and do not require RT or need escalated treatment. However, there is no consensus definition and optimum time point for response assessment. In advanced-stage patients, bilateral bone marrow aspirate and biopsies are also required for staging of pediatric HL. Surveillance scans are recommended after completion of therapy to identify early relapse in high-risk patients. The Childhood Hodgkin Lymphoma International Prognostic Score (CHIPS) system described four clinical factors that are predictive of worse event-free survival (EFS): stage IV disease, large mediastinal adenopathy, albumin level < 3.5 g/dL, and fever [35]. These findings provided the basis for prospective COG clinical trials. Most pediatric HL research groups also follow the presence of molecular and genetic factors for risk stratification, which informs treatment strategies and improves outcomes.

## MOLECULAR AND GENETIC PROGNOSTIC BIOMARKERS

Although prognostic biomarkers for pediatric HL have been investigated (Table 2), none have been sufficiently informative to be of significant value in clinical practice. Thus, molecular markers that can identify high-risk patients and predict risk of treatment failure are needed. Moreover, the availability of predictive biomarkers could aid the development of possible therapeutic targets that would help balance treatment outcomes and late effects of therapy. Several putative prognostic factors linked to pediatric HL are discussed below (also see Table 2).

**Table 2:** Prognostic biomarkers in pediatric Hodgkin lymphoma

Biomarker	Function	Patients (n)	Median/ mean Age (years)	Specimen type	Method	Levels/Expression pattern	Prognosis	Associated with	Ref
p53	cell cycle and apoptosis	54	8	FFPE	IHC	81% patients overexpressed	No significant difference	-	[37]
bcl-2	anti- and pro-apoptosis	104	14	TMA	IHC	52.4% patients positive	(↓) 0.8 fold 10 yr EFS (*P* = 0.048) in high risk	unfavorable risk	[36]
Ki-67	proliferation	121	8	pretreatment LN biopsy	IHC	100% cases nuclear positive expression	(↓) 0.7 fold 5 yr FFS in low vs. high PI (cutoff 74%, *P* = 0.016)	-	[43]
Ki-67	proliferation	224	13.7	biopsy	IHC	100% patients positive	No significant difference	No significant correlation	[41]
IL-10/IL-12	anti-inflammatory/pro-inflammatory	30 vs. 30 controls	15.4	pretreatment serum	ELISA	(↑) > 2 fold in IL10 and IL-12 levels in tumors vs. healthy control (*p* < 0.00001)	-	general symptoms	[44]
CD30 + cells	proliferation	96	14	TMA	IHC	45% cases positive with >5% cellularity	(↓) EFS in high vs. low CD30+ cells (cutoff 5%, *P* = 0.048)	-	[46]
sCD30	proliferation	303	-	pretreatment serum	ELISA	(↑) 78.2% patients (> 20 U/mL)	(↓) 0.6 fold EFS high vs. low CD30 levels (cutoff 100 U/mL, *P* < 0.001)	stage, B symptoms, tumor burden	[47]
ICAM-1 (CD-54)	cell-cell adhesion, cell-mediated cytotoxicity	69 vs. 32	14	pretreatment serum	ELISA	(↑) 2 fold in tumors vs. normal controls (*P* = .0001); (↓) in patients from diagnosis to CR (*P* < 0.0001)	(↓) DFS in high vs. low ICAM-1 levels (cutoff 500 ng/ml, P = 0.016)	advanced disease, B symptom, higher ESR, relapse	[51]
ICAM-1 (CD-54)	cell-cell adhesion, cell-mediated cytotoxicity	12 vs. 8 controls	7.4	pretreatment serum	ELISA	(↑) ∼7 fold in tumors vs. control (*p* < 0.000)	(↑) bad outcome (death) in high ICAM-1 levels (1,894.9 +/− 149.8 ng/ml, *P* = 0.009)	B symptoms, LDH levels	[52]
ICAM-1 (CD-54)	cell-cell adhesion, cell-mediated cytotoxicity	10 vs. 12 controls	-	pretreatment serum	ELISA	(↑) 2 fold in tumors vs. control (*p* < 0.01)	(↓) 0.4 fold 3 yr survival in high vs low ICAM-1 levels (median 286.4 ng/ml, *P* < .05)	high ESR	[50]
CD-44	metastasis	16 vs. 12 controls		FFPE	IHC	75% positivity in advanced stage	-	high serum levels	[54]
				pretreatment serum	ELISA	(↑) ∼2 fold in tumors vs. control (*p* < 0.001); (↓) in patients from diagnosis to CR (*P* < 0.05)	(↓) 0.2 fold 3 yr OS in high vs. low ICAM-1 levels (median 1627 ng/ml, *P* = 0.03)	high ESR, B-symptoms, advanced-stage disease	
CD-44	metastasis	18 vs. 20 controls	-	pretreatment serum	ELISA	(↑) ∼2 fold in tumors vs. control (*p* = 0.001)	-	LDH levels, advanced disease	[53]
α-1-antitrypsin	protease inhibitor	22	14.7	pretreatment serum	SELDI	(↑) 3.5 fold mean Intensity in stage IV sera vs. stage II	-	advanced stage	[56]
NK cells	immunosurveillance	38	8.5	tissue sections	IHC	Mean CD-57+ cell number 173.42; (↓) 2 fold in relapsed cases	(↓) EFS in low vs. high CD-57+ cells (cutoff 150, *P* = 0.0207).	-	[57]
NF-kB	lymphocyte proliferation and survival	102	15	pretreatment LN biopsy	IHC	(↑) cytoplasmic NF-κB2 in H/RS cells vs. controls	(↓) EFS in increased Rel-B (*P* = 0.009), NIK (*P* = 0.015), and A20 (*P* = 0.03) expression	slow response to therapy	[22]
Heparanase	metastasis and angiogenesis	19	-	pre and post treatment blood	ELISA	(↑) 6 fold Heparanase in patients vs. control; (↓) 1.7 fold at restaging (*P* = .035)	-	treatment response	[67]
VEGEF	angiogenesis	22 vs. 20 controls	13	pretreatment blood	ELISA	(↑) > 4 fold VEGEF in patients vs. controls (*P* = 0.0001)*	(↑) 5 fold unsuccessful treatment in high vs. low VEGEF level (cutoff 33.4 pg/ml, *P* = 0.01)*	-	[70]
VEGEF	angiogenesis	19	10.3	pre and post treatment blood	ELISA	(↑) > 6 fold plasma levels at diagnosis vs. controls (*p* < .0001)	-	treatment response	[66]
CD-68+ cells	survival and metastasis	74	-	TMA	IHC	100% cases >5% CD68+ macrophages; 86% cases >25% macrophages	-	EBV positivity	[74]
CD-163+ cells	survival and metastasis	100	14	TMA	IHC	CD68+ve cells number 290.81 cells/mm2; CD163+ve cells number 164.1 cells/mm2	(↓) PFS in EBV- cases with high CD163+ cells; (↓) 5 year OS in high vs. low CD163/CD8 ratio (cutoff 2, *P* = 0.005)	histological type: MC	[72]

Abbreviations : CR, Complete Remission; DFS, Disease-free Survival; EBV, Epstein-Barr Virus; EFS, Event Free Survival; ELISA, Enzyme-linked Immunosorbent Assay; ESR, Erythrocyte Sedimentation Rate; FFPE, Formalin-fixed, Paraffin-embedded; H/RS, Hodgkin and Reed Sternberg; HL, Hodgkin Lymphoma; IHC, Immunohistochemistry; ISH, *In Situ* Hybridization; LDH, Lactate Dehydrogenase; LN, Lymph Node; MC, Mixed Cellularity; NF-kB, Nuclear Factor Kappa-light-chain-enhancer of Activated B Cells; OS, Overall Survival; PFS, Progression Free Survival; RT-qPCR, Quantitative Reverse Transcription Polymerase Chain Reaction; SD, Standard Deviation; SELDI, Surface-enhanced Laser Desorption/Ionization; TMA, tumor associated macrophages

* Combined results of HL and NHL

### Bcl-2

B-cell leukemia/lymphoma 2 (*Bcl-2*) confers a protective advantage by mediating LMP-1-driven immortalization and enabling cells to evade programmed cell death. Approximately 50% of pediatric HL patients express Bcl-2 [36], and this Bcl-2 positivity is associated with decreased EFS in low-risk patients, and unfavorable risk patients in multivariate analysis [36]. However, some reports suggested that Bcl-2 expression did not affect outcomes [24, 37], and thus the prognostic value of Bcl-2 expression in HL is unclear.

### Ki-67

Several studies documented that high expression levels of the cell proliferation marker Ki-67 are common in HL [38–40] and can negatively impact outcome [40]. However, an immunohistochemical study of 224 pediatric patients enrolled in the German Society for Pediatric Oncology and Hematology (GPOH) HD-90 and HD-95 trials found that high Ki-67 expression in HRS cells as well as lymphocytic and histiocytic cells was not related to either advanced clinical stage or poor clinical outcome. Meanwhile, expression of the cell cycle marker repp86, which generally becomes detectable at the G1-S boundary, was low, implying that HRS or lymphocytic and histiocytic cells are arrested in the G1 phase of the cell cycle [41]. Better outcomes in pediatric HL cases that have a high proliferative index are attributed to increased susceptibility of proliferative cells to chemotherapeutic drugs [17, 42, 43]. Therefore, the role of Ki-67 and other cell cycle antigens in HL prognosis remains elusive and requires further investigation.

### IL-10 and IL-12

**Figure 1:**
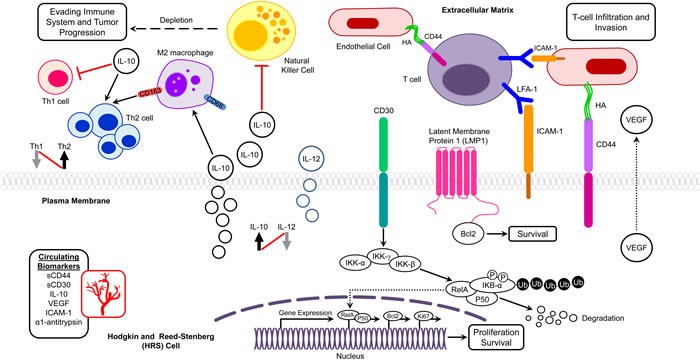
Potential molecular biomarkers in pediatric Hodgkin lymphoma Interplay of biomarkers in different molecular networks in the tumor microenvironment of Hodgkin lymphoma presenting with infiltration, evasion, and metastasis. Bcl-2, B-cell lymphoma 2; HA, Hyaluronic acid; ICAM-1, Intercellular Adhesion Molecule 1; IκB, Inhibitor of κB; IKK, IκB kinase; LFA-1, Lymphocyte Function Antigen-1; LMP-1, latent membrane protein 1; Th, T helper; VEGEF, Vascular Growth Endothelial Factor.

The Th2 cytokine IL-10 is expressed in about 30-50% of HL cases. IL-12 is involved in Th1 differentiation and is expressed in primary cHL tumors. IL-10 and IL-12 have antagonistic actions and shifts in Th1/Th2 cytokine ratios to Th2 predominance are common in cancer patients. (Figure 1). Elevated IL-10 and IL-12 levels in pediatric patients were found to have prognostic significance and were correlated with poor response to therapy, relapse, and shorter EFS and overall survival (OS) (Table 2) [44]. Additionally, IL-10/IL-12 ratios were significantly higher (*p* = 0.044) among symptomatic patients relative to asymptomatic patients, suggesting a role for these cytokines in HL.

### CD30

Overexpression of CD30 results in ligand-independent constitutive signaling that activates the transcription factors NF-κB and activator protein-1 (AP-1), which is critical for HRS cell survival (Figure 1) [45]. Under normal physiologic conditions, CD30 is not typically expressed on most human tissues and thus its selective expression on HRS cells makes it an optimal target for directed therapy. The association of high numbers of CD30+ RS cells with poor survival is suggestive of the potential prognostic value of CD30 expression in pediatric HL [46]. The extracellular region of CD30 is proteolytically cleaved from CD30+ cells, possibly on activation by CD30L, to produce a soluble form of CD30, sCD30, which can be detected in the serum. A study by Nadali *et al.* showed that the presence of sCD30 in HL at presentation correlates with several clinical features, including advanced stage, B symptoms, and tumor bulkiness [47]. They also found that sCD30 serum levels > 100 U/mL at diagnosis entailed a significantly higher risk of treatment failure [47]. A comparison of low-risk patients (stage I and II) with sCD30 < 100 U/mL to those of clinically advanced stage (III and IV) patients with sCD30 levels ≥ 100 U/mL, showed a 42% decrease in EFS for those patients with higher sCD30 levels (*P* < 0.001) (Table 2). The elevated sCD30 levels in HL reflect the functional behavior of HRS cells, and suggest that sCD30 may play a pathophysiologic role by mediating interactions between cytokines and the tumor microenvironment.

### Intercellular adhesion molecule-1 (ICAM-1, CD54)

ICAM-1 is involved in the development and progression of the malignant phenotype, and is overexpressed by HRS cells (Figure 1) [48, 49]. Serum ICAM-1 levels have been found to increase by 2-7 fold in pediatric HL patients, and levels decline or reach normal with complete remission (CR) (Table 2) [50, 51]. High serum ICAM-1 levels are also associated with advanced stages, B symptoms, higher ESR, relapses, and poor outcome [50–52]. Moreover, elevated ICAM-1 levels in patients with advanced stage disease may represent an increased host immune response to tumor cells or simply reflect a larger tumor burden.

### CD44

CD44 is a hyaluronic acid receptor that is involved in tumor development (Figure 1). Elevated serum sCD44 levels in pediatric HL patients are associated with B-symptoms, advanced stages, and poor survival [53, 54]. Additionally, higher expression levels of CD44 in tumor tissues are correlated with higher serum sCD44 levels, suggesting that at least a part of sCD44 found in HL patients originated from tumor cells [54].

### Alpha-1-antitrypsin (AAT)

AAT is a systemic inhibitor of neutrophil elastase (NE). An imbalance between AAT and NE may lead to tissue damage that results in tumorigenesis, invasion, and metastasis [55]. Alpha-1-antitrypsin was identified as a biomarker of tumor stage severity using surface enhanced laser desorption/ionization (SELDI-TOF) in 22 pediatric HL patients [56]. A fragment corresponding to 11.7 kDa identified as AAT was expressed to a greater extent in stage IV HL patients than in stage II patients (*P* = 0.03). An observed cluster of peaks was thought to represent allelic variants of AAT, which have been implicated in increased risk of multiple forms of cancer. The role of AAT in HL would benefit from additional analysis.

### Natural Killer (NK) cells

Due to their cytotoxic activity against tumors *in vivo*, NK cells have been evaluated as prognostic markers in different cancers (Figure 1). In an immunohistochemical analysis of NK cells in 38 pediatric HL patients, a significant decrease in EFS was found among patients with low CD57+ cell counts (NK cells) compared to those with high CD 57+ cell counts [57]. In contrast, another pediatric HL study (*n* = 17) found no correlation between NK cell activity and prognosis [58]. The difference in these results could be attributed to the different detection methods that were used to identify NK cells.

### Nuclear Factor-κB (NF-κB)

NF-κB proteins represent a family of inducible transcription factors that includes p65 (RelA), RelB, c-Rel, NF-κB1 (p105/p50), and NF-κB2 (p100/p52) [59]. These transcription factors mediate expression of genes involved in the immune response, cell proliferation, tumor metastasis, inflammation, and viral replication. NF-κB proteins form various homodimers and heterodimers, and are retained in an inactive form by cytoplasmic association with the IκB-α inhibitory protein [59]. Canonical and non-canonical pathways both lead to NF-κB activation, and both require activation of the IKK complex that consists of catalytic kinase subunits (IKKα/IKKβ) and the regulatory scaffold protein IKKγ [60].

The canonical NF-κB pathway is triggered by numerous signals, including those mediated by innate and adaptive immune receptors. Tumor necrosis factor (TNF-α) activates NF-κB via canonical phosphorylation of IKK that is mediated by the TNFRSF1A associated via death domain (TRADD), TNF receptor associated factor 2 (TRAF2), and NF-κB inducing kinase (NIK) [61]. When a cell receives a stimulatory signal, IκB-α is phosphorylated by IKK (IκB kinase) to induce IκB-α polyubiquitination and proteolytic degradation (Figure 1). Following IκB-degradation, NF-κB dimers translocate into the nucleus to activate gene transcription [62]. In the non-canonical pathway NF-κB2 (p100/RelB) complexes are inactive in the cytoplasm. The non-canonical pathway of NF-κB activation operates in response to the ligation of only certain TNFR superfamily members. In the non-canonical pathway, TRAF stimulates NIK, which subsequently activates IKK-α that in turn phosphorylates p100 leading to the processing and liberation of the p52/RelB active heterodimer [60].

Dysregulated NF-κB signaling leads to constitutive NF-κB activation, which is common in various lymphoid malignancies, and is a hallmark of HRS cells [62, 63]. In adult HL patients, studies have reported that NF-κB mutations promote constitutive activity of NF-κB signaling pathways [63, 64], but in pediatric HL, only a few studies have investigated the NF-κB pathway. The COG clinical trial AHOD0031 that examined intermediate-risk pediatric patient samples (*n* = 102) to clarify the role of NF-κB pathway proteins in HL found increased protein expression and nuclear localization of proteins in both the classical and alternative NF-κB pathways in pediatric patients compared to controls [22]. NF-κB pathway protein expression was dysregulated in EBV+ tumors and in patients with a slow early response to therapy. Five NF-κB proteins, including nuclear Rel-B, NIK, and A20, along with cytoplasmic Rel-A and IKK-β, were significantly associated with decreased EFS in multivariate analysis (Table 2). Notably, elevated levels of the non-canonical pathway protein NIK were also associated with a slow response to therapy (P = 0.005) [22]. In a phase II clinical trial of the proteasome inhibitor bortezomib (see below) in combination with ifosfamide/vinorelbine, nuclear phospho-RelA and RelB NF-κB subunits, as well as cytoplasmic RelB, were overexpressed in all relapsed HL patients compared to either non-malignant tissue or to HL patients at original diagnosis, but expression of these proteins was not associated with EBV status [65]. These findings suggest that the NF-κB pathway plays an essential role in pediatric HL high-risk patients.

### Heparanase

Heparanase is an endoglycosidase that cleaves heparan sulfate proteoglycans (HSPG) to alter the structure of the extracellular matrix (ECM), and also plays an important role in tumor metastasis. The prognostic significance of heparanase in pediatric HL patients was demonstrated by a 6-fold increase in heparanase level at diagnosis and a subsequent decrease associated with complete remission (CR) or a good partial response (Table 2) [66, 67]. Heparanase levels remained stable in patients with poor treatment response and tumor progression, indicating its association with tumor burden.

### Vascular growth endothelial factor (VEGF)

VEGF is produced by cancer cells, and its activation promotes proliferation and migration of endothelial cells with formation of new blood vessels. High levels of circulating VEGF are a well-established indicator of poor prognosis [68]. Serum VEGF levels have been reported to be elevated by 4-6 fold in pediatric HL patients compared to normal controls (*P* < 0.0001) (Table 2) [69, 70]. VEGF levels in 9 children correlated with the treatment response assessed after two cycles of chemotherapy, and post-therapy levels were decreased by 50% in children who were in good partial remission or CR [69]. A similar analysis revealed that the median VEGF concentration in the group with unsuccessful treatment (partial remission, progressive disease, and early relapse) was significantly higher than that in the subgroup of children who achieved CR (*P* = 0.02) [70]. Baseline serum VEGF concentrations among pediatric HL patients thus appear to be a promising predictive marker of response to treatment.

### Tumor associated macrophages

In HL, scant HRS cells (1% of the cell population) are surrounded by a dense microenvironment consisting of a variety of reactive cells (99% of the cell population) that include T cells, B cells, plasma cells, macrophages, mast cells, dendritic cells, neutrophils, eosinophils, and fibroblasts. HRS cells attract infiltrating cells by secreting cytokines and chemokines that in turn play a role in HRS cell survival, proliferation, and inflammatory reaction [12]. HRS cells also secrete lymphotoxin-α (LTα), which stimulates endothelial cells to upregulate ICAM-1 and hyaluronan levels to enhance recruitment of T cells into the tumor milieu [71]. Recruitment of infiltrating cells is also stimulated by reactive cells- particularly macrophages and mast cells. In a Th2-shifted immunosuppressive tumor microenvironment, HRS cells are rescued from attack by Th1 cells, which consequently affect HL pathogenesis and prognosis (Figure 1).

The prognostic impact of tumor associated macrophages (TAMs) was assessed by Barros *et al*. and Gupta *et al*. in 100 and 96 pediatric HL samples, respectively [46, 72]. In contrast to the HL study of adult patients by Steidl *et al*. [73], neither of the two studies found any significant impact of CD68+ macrophages on disease outcome in pediatric populations. In another study, a high number of CD68+ cells in the tumor microenvironment of patients, which may be associated with the presence of EBV, was associated with a good prognosis [74]. However, Barros *et al*. showed that high numbers of CD163+ macrophages were associated with worse progression-free survival in EBV- cases but not in EBV+ cases (Table 2) [72]. These results indicate that the composition of TAMs in the tumor microenvironment is distinct in pediatric HL patients. In EBV+ cases, these macrophages are M1 polarized and therefore may mediate effective immune surveillance [75]. Further studies that use a combination of tumor milieu components in a comparable series of pediatric HL patients may be helpful to understand the role of TAMs in tumor progression and prognosis. The role of EBV and age as potential confounding variables should also be investigated.

A Comparison of the results of biomarker studies in pediatric HL patients indicates that NF-κB, sCD30, and ICAM-1, are most likely to be independent prognostic markers for clinical outcome predictions (Table 2), although ICAM-1 requires validation with a larger cohort. A study by Horie *et al*. [45] revealed that CD30 overexpression in HRS cells results in ligand independent signaling and constitutive NF-κB activation. The study also demonstrated that an adenovirus vector carrying either a decoy CD30 that lacks the cytoplasmic region or a dominant negative IkBα mutant could block NF-κB activation and induce apoptosis. Therefore, therapeutic approaches that focus on blocking CD30 and downregulating NF-κB expression could be effective therapeutic approaches for pediatric HL. Assessing levels of a combination of biomarkers such as NF-κB, sCD30, and ICAM-1 in pediatric HL patients would be helpful to gain insight into disease progression throughout the course of tumor management. A COG study (NCT01366157/AHOD11B1) is underway to assess the correlation between human GC-associated lymphoma (HGAL) protein levels and early response in low-risk pediatric HL. A comparison of HGAL protein markers in low-risk and intermediate-risk HL might provide insights into the early transforming events that lead to tumorigenesis.

Other potential biomarkers that have been investigated in adult HL are Thymus and activation-regulated chemokine (TARC), Syndecan-1 (SDC1), and Fibroblast growth factor-2 (FGF2). In adult HL, TARC correlates with disease stage, tumor burden, PET positivity, and poorer outcome, which makes it an ideal HL biomarker [76–78]. SDC-1 expression on HRS cells ranges from completely negative to 45%-100% [2, 79, 80]. Increased serum levels or expression of SDC-1 have been associated with poor outcome and adverse prognosis [81, 82]. Fibroblast growth factor-2 (FGF2) has been shown to be overexpressed in putative circulating CD15+/CD30+ cells from poor outcome HL patients [81]. The levels of FGF2 and SDC1 were 245- and 91-fold higher, respectively, in the poor outcome group compared to the good outcome group [81]. However, in pediatric patients the prognostic significance of TARC, SDC-1, and FGF2 expression has not been evaluated. Therefore, it may be useful to extrapolate the findings from adult HL patients to pediatric HL patients to help improve clinical management of high-risk patients in particular.

### Genetic polymorphism

Single nucleotide polymorphisms (SNPs) within the promoter regions of cytokine genes have been associated with differential transcription levels, and may have opposing pleiotropic effects in different cancer types. Clinical and pathological features of cHL often result from cytokine imbalances that produce aberrant immune responses. Due to their effects on cytokine expression, cytokine promoter region SNPs are likely mediators of inherited risk. In an analysis of SNPs in various cytokines among 37 pediatric HL patients, the CD14-159 (C>T) SNP was suggested to be associated with pediatric HL [83]. Soluble CD14 (sCD14) is a regulatory molecule that modulates cellular and humoral responses by interacting with T and B cells [84]. Notably, CD14 is located in the same cluster on chromosome 5q31 as other Th2 cytokines, suggesting that CD14 gene polymorphisms may be involved in regulating Th2-type responses [85]. This possibility highlights the importance of a Th2 biased tumor microenvironment. In another example, a polymorphism in the untranslated region (UTR) of IL-12 (1188 3′ UTR A>C) is associated with a significant (2.8-fold) increase in risk in adolescent HL patients [86]. Moreover, IL-12 levels were lower in probands and their unaffected twins compared to controls, suggesting that decreased IL-12 levels are a potential risk factor for HL. Also, germline mutations in IL-2 inducible T cell kinase (ITK) reportedly cause EBV-associated lympho-proliferation. The novel homozygous nonsense mutation C1764G in ITK exon 16 found in 3 HL patients in a single family was shown to be a potential candidate for a heritable risk factor for EBV-associated HL [87]. Another study identified two heterozygous mutations, C49T and 922delG, in ITK genes in a pediatric patient who presented with B-cell hyperplasia followed by EBV-associated HL [88]. ITK is involved in NK cell maturation and survival, and plays a pivotal role in EBV infection responses. Mutations in the ITK gene lead to production of a truncated protein that produces aberrant immune system responses with severe clinical presentation. Although these findings suggest that ITK mutations may serve as a marker for EBV-associated HL in pediatric patients, how these mutations influence clinical and histopathological features of pediatric EBV-associated HL is currently under investigation (NCT01490801).

HL survivors are at significant risk for developing secondary malignancies. Two independent genome wide association studies (GWAS) reported that variants at chromosome 6q21 in pediatric HL patients were associated with radiation induced second malignant neoplasms (SMNs) [89, 90]. Best and colleagues revealed significant associations between SNPs on chromosome 6q21, rs4946728 (*P* = 0.002), and rs1040411 (*P* = 0.03) and the odds of developing SMNs, which increased by >3-fold and >2-fold per copy of the major allele rs4946728 and rs1040411, respectively [89]. These SNPs were also associated with decreased basal expression of the transcriptional repressor PRDM1 (PR domain containing 1, with ZNF domain, also known as BLIMP1) and impaired induction of PRDM1 expression after radiation exposure, suggesting a novel role for PRDM1 as a radiation-responsive tumor suppressor.

The glutathione S-transferase M1 (GSTM1) gene is polymorphic in humans. The frequency of the GSTM1 null genotype is approximately 50% in the U.S. and Europe. A childhood cancer survivor study (CCSS) of 650 childhood HL survivors in the U.S. and Canada demonstrated that individuals lacking GSTM1 have a significantly higher risk of developing SMN [91]. However, the magnitude of this effect was small, indicating that multiple genes may mediate genetic susceptibility to DNA damage. Therefore, additional studies will be needed to strengthen the associations of these putative risk factors with risk of SMN.

## TREATMENT

The standard chemotherapy regimen of MOPP (nitrogen mustard (mechlorethamine), vincristine, procarbazine and prednisolone) provided high therapeutic success rates in the 1970s. However, two decades later adverse complications became apparent, including risks of secondary malignancy, gonadal toxicity, and sterility in females because of the inclusion of an alkylating agent (mechlorethamine) in this regimen. The combination of ABVD (adriamycin (doxorubicin), bleomycin, vinblastine, and dacarbazine) proved to be more effective than MOPP and had a lower incidence of subsequent leukemia and infertility, and eventually replaced MOPP as the standard of care in the United States. However, ABVD therapy has its own set of complications, including cardiopulmonary toxicity caused by doxorubicin and bleomycin that are subject to the cumulative dose of chemotherapeutic agents and addition of RT. To reduce the risk of long-term toxicities in younger patients, pediatric HL treatment regimens have modified from those used to treat adult HL. Contemporary therapeutic approaches for pediatric HL are based on the refinement of risk group stratification that titrate the length and intensity of chemotherapy, as well as radiation dose using response assessment made through interim or post-chemotherapy PET/CT analysis.

### Treatment for favorable-risk pediatric patients

Various European and North American study groups used ABVD derivatives such as ABVE (doxorubicin, bleomycin, vincristine, etoposide) [92], OEPA (vincristine, etoposide, prednisone, and doxorubicin) for boys, OPPA (vincristine, procarbazine, prednisone, and doxorubicin) for girls [93], and VAMP (vinblastine, doxorubicin, methotrexate, and prednisone) in an effort to limit the toxicities caused by doxorubicin, bleomycin and procarbazine [94]. Based on the response to these contemporary multi-agent chemotherapy regimens, patients could also be optionally treated with low dose (15-30 Gy) involved field radiation (IFRT), which provided an EFS >90% and overall survival (OS) >95% without significant radiation or alkylator toxicity (Table 3). The occurrence of complications from high-dose RT such as cardiovascular disease, second malignancy, hypothyroidism, infertility, and impaired musculoskeletal development in pediatric survivors [95–97] motivated groups to evaluate treatment methods that omit radiation altogether for low-risk patients. Indeed, several clinical trials, evaluating response based chemotherapy by computed tomography, magnetic resonance imaging, gallium scans, and PET scans, demonstrated that radiation therapy may not be needed for low-risk patients who achieve CR after 2 cycles of chemotherapy (Table 3) [94, 98, 99]. The CCG 5942 randomized trial was the first study in children and young adults with HL that evaluated the effect of omitting RT for patients who achieved a complete response to initial COPP/ABV chemotherapy [99]. The long-term outcome of this study showed a 10-year EFS rate of 91.2% for the IFRT group and 82.9% for the no further therapy group (*P* = 0.004). However, the overall survival probability was similar in both randomization arms (*P* = 0.50) (Table 3). In a study of favorable risk patients, VAMP chemotherapy, which eliminates the alkylating agents, epipodophyllotoxins and bleomycin, resulted in a 5-year EFS of 89%, with no significant difference in outcome between those who were treated with RT (93%) and those who were not (89%) (*P* = 0.61), and no patient developed SMNs. However, this finding awaits long-term validation in larger cohorts [94] (Table 3). In the GPOH-HD95 trial [98], the 10-year PFS rates in low-risk patients were not statistically different for the group without RT (97.0%) compared to the RT group (92.2%) (*P* = 0.21), and the OS rates in all treatment groups were also promising and similar. However, the 10 year PFS rates were unsatisfactory for intermediate and advanced stage disease patients who did not receive RT. The COG trial used Rapid Early Response as a measure of chemosensitivity, which has been a promising predictor of EFS [100]. PET-guided early interim imaging is also a promising approach to reduce treatment-related toxicities among patients undergoing chemotherapy [101, 102]. The results of these studies indicate that reduced RT and modified chemotherapy regimens (cumulative doses of anthracyclines and alkylating agents) produced fewer complications, and are workable strategies to develop balanced treatment protocols through which favorable-risk patients can avoid late sequelae. Of note, the latency and frequency of secondary tumors is comparable in children treated with low dose (15-25 Gy) and high-dose IFRT [103]. Taken together, these findings appear to justify the omission of RT in early stage pediatric HL patients.

**Table 3:** Summary of clinical trials in low, intermediate, and high risk pediatric Hodgkin lymphoma

Trial Identifier	Pts. (n)	Years of Study	Chemotherapy	RT (Gy)	Risk groups	EFS, PFS %	OS %	Toxicity, RR patients (%)	Ref
NCT00025259/AHOD0031 (III)	1712	2002-2012	4 ABVE-PC ± 2 DECA (SER patients)	RER: CR no RT; SER: 21, IF	Intermediate risk/high risk IB, IAE, IIB, IIAE, IIIA, IVA bulk/no bulk; IA, IIA with bulk	4 yrs 85.0 4yrs 86.9 RER	4 yrs 97.8 4 yrs 98.5 RER	hematologic (83.8); febrile neutropenia (24.9); RR patients (14.1); SMN (0.8)	[102]
						4 yrs 77.4 SER (P < 0.001)	4 yrs 95.3 SER (P < 0.001)		
GPOHHD-95	1018	1995-2001	2 OPPA (girls, Group I) 2 OEPA (Boys Group II) Additional 2-4 COPP for Group 2 and 3	CR: No RT 20 >75% tumor regression 30 <75% tumor regression	Low Risk (Group I: IA/B, IIA)	10 yrs PFS 93.2	10 yrs 98.8	RR patients (5.99); SMN (1.86)	[98]
					Intermediate/high risk (Group 2: IIB, IIIA, IAE, IBE, IIAE)	10 yrs PFS 85.5	10 yrs 95		
					high risk (Group 3:IIIB, IV, IIBE, IIIAE, IIIBE)				
NCT00145600/HOD99 (II)	88	2000-2008	4 VAMP	CR: no RT; PR: 25.5, IF	low I and II	2 yrs 90.8	2 yrs 100	Neutropenia (60)	[94]
NCT00002827/P9426	255	1996-2000	2-4 ABVE ±DRZ	25.5, IF	low I, IIA,IIIA	8 yrs 86.0 85.7 no DRZ 86.8 DRZ (P=0.70)	8 yrs 97	no DRZ (54); DRZ (68.8) Neutropenia; RR patients (11.4); SMN (1.96)	[152]
CCG-5942	826	1995-1998	Group 1 : 4COPP/ABV	CR: no RT, PR: 21, IF	Low risk (Group 1:I and II)	10 yrs 89.1 no RT 100 RT	10 yrs 95.9 no RT 97.1 RT	RR patients (13.1); SMN (0.36)	[99]
			Group 2: 6COPP/ABV		Intermediate Risk (Group 2: I, II, III)	10 yrs 78.95 no RT 86.25 RT			
			Group 3: 6COPP/ABV		High Risk (Group 3: IV)				
NCT00004010/COG-59704	98	1999-2003	4 BEACOPP2 (all patients), RER: 4 COPP/ABV (Females), 2 ABVD (Males) SER: 4 BEACOPP2	RER: Females no RT, male 21, IF SER: both females and males 21, IF	High risk IV, II/III with B symptoms and bulk	5 yrs 94	5 yrs 97	Thrombocytopenia (62); Neutropenia (83); RR patients (3.0); SMN (2.0)	[105]
GPOH-HD-2002	573	2002-2005	2 OEPA (Boys) 2 OPPA (Girls)	CR: no RT	Low Risk: (Group 1: IA, IB, IIA)	5 yrs 92.0	5 yrs 99.5	OEPA (70.5)/ OPPA (52.4) Leukopenia; OEPA (81.5)/ OPPA (57.1) Neutropenia; RR patients (1.74); SMN (1.9)	[93]
			2 OEPA-COPDAC (Boys) 2 OPPA-COPP (Girls)	19.8-35, IF	Intermediate Risk: (Group 2: IE, IIB, IIAE, IIIA)	5 yrs 87.7	5 yrs 96.2		
			4 OEPA-COPDAC (Boys) 4 OPPA-COPP (Girls)		high risk: (Group 3: IIBE, IIIAE, IIIB, IVA, IVB, IVE)				
NCT00005578/P9425	216	1997-2004	5 ABVE-PC DRZ	21	Intermediate Risk: IB, IIA/IIIA1,IIIA2 High risk: IIB, IIIB, IV	5 yrs 84.3	5 yrs 95.4	no DRZ (29.6/ DRZ (72.6) Thrombocytopenia; no DRZ (77.8)/ DRZ (93.4) Neutropenia; no DRZ (0.9), DRZ (2.8) RR patients (11.5)	[100]

ABVD, Doxorubicin, Bleomycin, Vinblastine, and Dacarbazine; ABVE, Doxorubicin, Bleomycin, Vincristine, and Etoposide; BEACOPP, Bleomycin, Etoposide, Doxorubicin, Cyclophosphamide, Vincristine, Procarbazine, Prednisone; COPDAC, Cyclophosphamide, Vincristine, Prednisone, and Dacarbazine; COPP, Cyclophosphamide, Vincristine, Procarbazine, and Prednisone; CR, Complete Remission; DECA, Dexamethasone, Etoposide, Cisplatin, and Cytarabine; DRZ, Dexrazoxane; IF, Involve Field; OEPA, Vincristine, Etoposide, Prednisone, and Doxorubicin; OPPA, Vincristine, Procarbazine, Prednisone, and Doxorubicin; OS, Overall survival; PFS, Progression Free Survival; PR, Partial Remission; RER, Rapid Early Response; RR, Relapsed/Refractory; RT, Radiotherapy; SER, Slow Early Response; SMN, Second Malignant Neoplasm; VAMP, Vinblastine, Doxorubicin, Methotrexate, and Prednisone

### Treatment for intermediate/high-risk pediatric patients

Many pediatric oncology groups consider that the combined chemotherapy and RT modality for high-risk patients is superior to chemotherapy-only protocols as a means to avoid reaching the toxicity threshold for high cumulative doses of alkylating agents, bleomycin, and anthracyclines, as well as to reduce relapses and subsequent need for toxic salvage therapy. Adjuvant RT has been demonstrated to be effective in many clinical trials for pediatric high-risk HL patients. The Stanford/St. Jude/Dana Farber consortium investigated VAMP/COP and response-based IFRT for high-risk HL patients [104]. The poor outcomes of the study indicated that limited cumulative doses of alkylating agents and anthracycline chemotherapy in combination with low-dose IFRT are not sufficient for disease control in high-risk patients. The incorporation of dose-intensification strategies such as BEACOPP (bleomycin, etoposide, doxorubicin, cyclophosphamide, procarbazine, and prednisone) [105] and ABVE-PC [102] (doxorubicin, bleomycin, vincristine, etoposide, prednisone, and cyclophosphamide) in intermediate/high-risk patients have shown promising outcomes, although BEACOPP has not been widely used because of its various toxicities (e.g., hematologic toxicity, cardiopulmonary toxicity, secondary malignancy, and infertility) (Table 3). However, an escalated BEACOPP regimen in adult HL patients was recently evaluated using PET-guided interim response assessment to identify poor outcome patients and direct subsequent treatment modifications [106]. In this study, all patients received two cycles of escalated BEACOPP, then underwent PET imaging. In the PET arm, patients who were PET-positive received four additional cycles of escalated BEACOPP and PET-negative patients received four cycles of ABVD. In the standard arm, patients received 6 cycles of escalated BEACOPP regardless of PET status. The estimated 2-year PFS was similar in both groups: 91.6% in the standard arm and 88.3% in the PET-driven arm (*p* = 0.79). Future studies will be needed to examine the feasibility of this strategy for pediatric patients with PET-positive advanced stage HL. The ABVE-PC regimen excludes procarbazine and limits the cumulative dose of epipodophyllotoxins and anthracyclines, thereby lowering the number of SMNs (0.8%) [102]. This regimen was used in a COG trial where it showed excellent 4 year EFS (85%) and OS (98%), although the relapse rate was 14% (Table 3). Pediatric Oncology Group used this dose-dense regimen in its P9425 trial with or without dexrazoxane (topoisomerase inhibitor), as a cardioprotectant to prevent anthracycline-associated cardiac toxicity during treatment. The study in advanced HL patients proved to be effective in disease control with a 5-year EFS of 84% and OS of 95%, however dexrazoxane could possibly be associated with increased risk of second malignancies and acute myeloid leukemia (AML)/myelodysplastic syndrome when used with ABVE-PC regimen [107]. The study evaluating long-term effects of dexrazoxane on secondary cancer and cardiac health among childhood HL survivors is needed to validate these results. In the GPOH-HD-2002 trial, gender-stratified chemotherapy with OEPA-COPDAC for boys and OPPA-COPP for girls was used to treat intermediate and advanced stage HL patients. In this trial, procarbazine in COPP was replaced by dacarbazine, which has reduced gonadotoxicity and has seen wide use in ABVD regimens. Response based IFRT was an integral part of the treatment for high-risk groups, even in patients who achieved CR after chemotherapy. The trial showed 5-year EFS of 89% and OS of 97.4% with SMNs occurring in ∼ 2% of cases. Both regimens were found to be interchangeable among male and female pediatric patients with intermediate and advanced stage cHL (Table 3) [93]. The Euronet trial (NCT00433459) is currently underway to compare the efficacy OEPA-COPDAC and OEPA-COPP treatments using PET-guided response assessment after two cycles of chemotherapy. In light of these clinical trial results, the challenge is to prevent relapse in early stage patients who did not receive RT and to limit the dose of alkylating agents and RT to minimize the incidence of SMNs.

### Salvage therapy for relapsed/refractory (RR) pediatric patients

Although there has been remarkable progress in the treatment of pediatric HL that resulted in high cure rates, 10-25% of cases are relapse/refractory (RR) patients, which present greater challenges for clinical management. There is currently no gold standard for second line treatment, but the retrieval regimen for RR patients includes re-induction or salvage chemotherapy and, thereafter, high dose chemotherapy (HDCT) followed by autologous stem cell transplantation (ASCT). This regimen is associated with remission rates of approximately 50-65% [108, 109]. However, low-risk patients with late relapse and limited stage may be retrieved with standard dose chemotherapy (SDCT) and RT. The most common prognostic factors are chemosensitivity to initial salvage therapy [110], primary progressive disease [111, 112], time to relapse [113], and extranodal disease [114]. As with adult patients, pediatric patients also show poor prognosis, associated with time from diagnosis to first relapse of <1 year [115]. Likewise, primary progressive HL that remains refractory has a poor outcome with HDCT/ASCT in children. Therefore, alternative treatment approaches, including allogeneic stem cell transplant (alloSCT) or novel biological and targeted therapies, should be considered for these patients. In recent years, patients were more often retrieved with mini-BEAM [BCNU (carmustine), etoposide, cytarabine, melphalan] (BEAM chemotherapy in lower doses) [116]; MINE (mitoguazone, ifosfamide, vinorelbine, and etoposide) [113], or alternating IEP-ABVD (ifosfamide, etoposide, prednisolone) [109]. Reinduction regimen, ICE (Ifosfamide, carboplatin, and etoposide) in RR pediatric and adult patients has shown a combined response rate of 88% [117]. However, ICE poses a risk for myelosuppression and secondary malignancy because of alkylating agents and epipodophyllotoxins. Contemporary non-etoposide retrieval regimens in RR patients, such as the combination of ifosfamide with vinorelbine (IV) [118] or gemcitabine in combination with vinorelbine (GV) [119] resulted in overall response rates of 72-76% (Table 4). However, pediatric patients with early relapse or inadequate response rate are unlikely to achieve long-term remission with standard salvage therapy, and their survival remains very poor, ranging from 18-41% [109, 110, 115]. This low survival rate presents a therapeutic challenge for treating RR patients, and reinforces the need to test novel immune and non-immune strategies to combat HL.

### Targeted therapies

Due to the limited success rates of HDCT with ASCT, and the association of these regimens with significant risk of secondary malignancies, there is a need to develop alternative therapeutic approaches that are efficacious and safe for RR pediatric patients. Novel therapeutic approaches include the use of monoclonal antibodies, signal transduction inhibitors, immunotherapy, epigenetic agents such as histone deacetylase (HDAC) inhibitors and demethylating agents, and agents that target the tumor microenvironment [120]. Unfortunately, randomized clinical trials of these approaches have not yet been conducted in pediatric patients.

**Figure 2:**
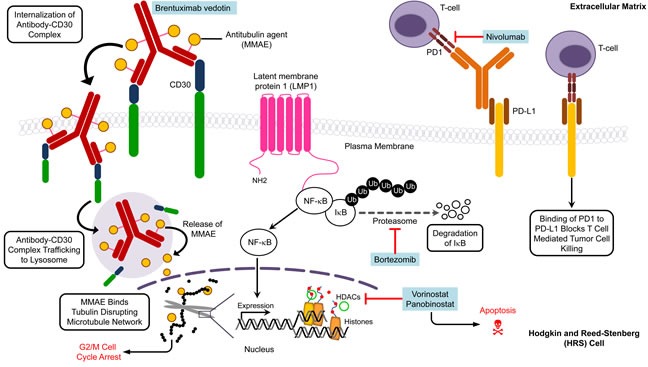
Molecular targets and agents affecting specific targets in pediatric Hodgkin lymphoma Multiple pathways are implicated in Hodgkin lymphoma and thus present potential targets for therapy. The various targets and agents illustrate the need for future clinical trials to focus on synergistic action of inhibitors to kill tumor cells. The drugs are listed in blue boxes adjacent to the corresponding target. HDAC, histone deacetylase; MMAE, monomethyl auristatin E; PD1, programmed cell death protein 1; PD-L1, programmed death-ligand 1.

Brentuximab vedotin (Bv) (SGN-35) is an anti-CD30 antibody-drug conjugate that delivers the anti-tubulin agent monomethyl auristatin E (MMAE) to HRS cells where it binds tubulin to disrupt the microtubule network and induce apoptosis [121] (Figure 2). Compared to standard salvage regimens, Bv alone (as a primary salvage chemotherapy) or combined with standard therapy (as a first line therapy in treatment-naive patients) produced better outcomes (ORR ∼ 65%) and reduced toxicity in RR patients [122, 123]. In a phase I/II trial, 16 pediatric patients with RR HL were treated with Bv to achieve ORR (CR+ PR) of 64% and 21% CR. These preliminary treatment results provide hope that Bv treatment may also benefit RR pediatric HL patients. Using the same guidelines as for adult patients, several contemporary pediatric clinical trials are underway to test the effectiveness of Bv as a front-line treatment: i) Bv substituting for vincristine in the OEPA/COPDAC regimen (NCT01920932); ii) Bv in combination with AVEPC or ABVE-PC alone (NCT02166463/AHOD1331 (Table 4). Also, the combination of Bv with gemcitabine is being assessed in a phase 1/2 trial for RR pediatric and young adult HL (NCT01780662/AHOD1221). The objective of these approaches is to reduce toxicity by using a combination of agents that have distinct mechanisms of action against tumor cells.

Proteasome inhibitors have been used to treat HL based on their ability to block degradation of IκBα that in turn inhibits NF-κB activation. Bortezomib (Velcade, PS341) selectively inhibits the 26S proteasome to stabilize proteins that are degraded by the ubiquitin-proteasome system, including the NF-κB inhibitor IκB [124] (Figure 2). The results from a phase 2 clinical trial (AHOD0521) investigating the tolerability and efficacy of bortezomib in combination with ifosfamide and vinorelbine (IV) in 23 evaluable RR pediatric HL patients demonstrated an ORR approaching 83%, which is a marked improvement over the poor response seen with this combination in adult HL patients (Table 4) [65, 125].

**Table 4:** Summary of completed and ongoing clinical trials involving relapsed/refractory pediatric Hodgkin lymphoma

Trial Identifier	Phase, status	Agent (trade name)	Manufacturer	Patients (N)	Response (%)	Ref
NCT00070304/ AHOD0321	II, completed	Gemcitabine (Gemzar), Vinorelbine (Navelbine)	Eli Lilly	30	76% (1 yr EFS 59.5%; OS 86.0%)	[119]
NCT00006760/ AHOD00P1	II, completed	Ifosfamide (Ifex), Vinorelbine (Navelbine)	Bristol-Myers Squibb; Pierre Fabre Pharmaceuticals, Inc.	66	72% (5 yr EFS 57.2%; OS 73.9%)	[118]
NCT00381940	II, completed	Bortezomib (Velcade)	Millennium	26	83	[65]
NCT01492088	I/II, ongoing/recruiting	Brentuximab Vedotin (Adcetris; SGN-35)	Seattle Genetics	16	64	[153]
NCT01920932	II, ongoing/ recruiting	Brentuximab Vedotin (Adcetris; SGN-35)	Seattle Genetics	-	-	
NCT02166463	III, ongoing/recruiting	Brentuximab Vedotin (Adcetris; SGN-35)	Seattle Genetics	-	-	
NCT00994500/ ADVL0916	I, completed	Vorinostat (Zolinza); Bortezomib (Velcade)	Merck	-	-	
NCT01321346	I, completed	Panobinostat/LBH-589 (Farydak)	Novartis	-	-	
NCT01748721	I, completed	MORAb-004 (Ontuxizumab)	Morphotek	-	-	
NCT02304458	I/II, ongoing/recruiting	Nivolumab	Bristol-Myers Squibb	-	-	

EFS, Event Free Survival; HDAC, Histone Deacetylase; NF-kB, Nuclear Factor Kappa-light-chain-enhancer of Activated B Cells; OS, Overall Survival; PD-L1, Programmed Death-ligand 1; TEM1, Tumor Endothelial Marker 1

To date, Bv and bortezomib have been shown to be the most effective agents to treat RR pediatric patients (Table 4) and highlights the essential role of the CD30 and NF-κB biomarkers in HL. The combination of Bv and Bortezomib may exert a synergistic cytotoxic effect that promotes downregulation of NF-κB expression (see Biomarkers section), and may represent a novel therapeutic approach to improve CD30-based targeted therapy. Moreover, an *in vitro* and xenograft model study revealed the synergistic cytotoxic effect of 5F11 (anti-CD30 mAb) and bortezomib in HRS cells, which again emphasizes the importance of CD30 in particular as a HL biomarker [126].

The HDAC inhibitors vorinostat and panobinostat have favorable antiproliferative activity that is manifested through cell cycle arrest and apoptosis (Figure 2). Interestingly, the anti-tumor activity of vorinostat is associated with decreased production of Th2 cytokines and chemokines, including TARC, that confers immunosuppression and protects tumor cells from cytotoxic T cells [127]. Therefore, vorinostat may sufficiently alter the tumor microenvironment to restore the Th1 anti-tumor response, and consequently suppress tumor progression and metastasis in HL patients. Panobinostat also inhibits T cell PD-1 expression in HL cell lines and RR HL patients, suggesting that this agent may promote tumor cell recognition and elimination by effector lymphocytes [128]. However, the clinical use of vorinostat and panobinostat as single agents has been of limited value in adult HL patients, and produced response rates of only 4% [129] and 27% [130], respectively. Thus, contemporary clinical trials are exploring their use in combination with other standard salvage agents in RR pediatric HL patients (NCT00994500; NCT01321346). HDAC inhibitors have been shown to interact synergistically with proteasome inhibitors to induce apoptosis [131], and studies are also underway to evaluate these findings in patients (Table 4) since combination therapies involving HDAC inhibitors and proteasome inhibitors may provide meaningful clinical outcomes.

Ontuxizumab (MORAb-004) is a recently developed humanized recombinant mouse antibody (Ab) directed against endosialin (TEM-1, CD248) [132]is predominantly expressed on the surface of cells with a mesenchymal origin, including tumor vasculature, tumor pericytes, and active tumor stromal fibroblasts [133, 134]. Inhibiting endosialin by monoclonal Ab can decrease tumor growth and tumor metastasis [135]. As such, clinical trials aimed to determine safety profiles and the optimal dose for endosialin-targeted drugs are underway in RR pediatric HL patients (NCT01748721).

Nivolumab (BMS-936558) is a monoclonal antibody that may block the PD-1 receptor expressed on peritumoral T cells. Programmed death-ligand 1 (PD-L1) is aberrantly expressed on HRS cells and interacts with PD-1 to contribute to evasion of immune detection by inhibiting T cell receptor signaling (Figure 2). Promising results from an early study in adult patients [136] prompted further investigation of nivolumab in RR pediatric HL patients (NCT02304458).

## SECOND MALIGNANT NEOPLASMS (SMNS) - FOCUS ON SECONDARY LEUKEMIA

SMNs have long been known to be a major complication in long-term survivors of HL. SMNs have a serious impact, particularly in children who, at the time of treatment, are still in the growth phase and thus are more sensitive to mutagenic compounds and radiations than adults [137, 138]. Among all childhood cancer survivors, HL survivors exhibit the highest risk for SMNs, with an absolute SMN excess of 5 to 8 per 1,000 person years of follow-up [139–141]. HL survivors are at increased risk for developing secondary sarcomas and carcinomas including breast, thyroid, gastrointestinal, lung, and non-Hodgkin lymphoma (NHL) caused by RT that can occur 10-20 years after HL therapy [139, 140]. HL survivors are also more susceptible to secondary leukemia that is attributed to alkylating agents (mechlorethamine and procarbazine) or epipodophyllotoxin (topoisomerase II inhibitor- etoposide) [142].

Table 5 lists the cumulative incidences, SIR values, and mortality rates for secondary leukemia among childhood HL survivors. Notably, up to 37% of secondary malignancies that occur after HL treatment are leukemias, and of these, 70-90% are AML (Table 5). In CCSS, secondary AML affected 1.8% of HL patients, but only those cases who survived at least 5 years after primary cancer diagnosis, while leukemia can emerge as early as 2 years after HL treatment [141]. The overall cumulative incidence of secondary leukemia up to 30 years after treatment for childhood HL ranges from 0.8% to 2.1%, depending on the therapy (Table 5). The risk of developing leukemia peaks at 4 to 6 years of follow-up, declines over the next 10 years, and then plateaus at around 15-20 years [139, 140]. However, the latency period between initiating treatment with topoisomerase II inhibitors and leukemia onset is even shorter, with a median of 2 to 3 years [143]. The risk of developing secondary leukemia, including AML, following childhood treatment for HL is 10.4- to 174.8-fold greater than the risk for the general pediatric population (Table 5). However, variability in cohort size, patient classification, treatment approach, and duration of follow-up complicates the comparison of these studies. Dorffel *et al*. reported relatively lower standardized incidence ratio (SIR) value for secondary leukemia apparently because none of the patients in this cohort were given the alkylating agent mechlorethamine in chemotherapeutic DAL/GPOH protocols for HL (Table 5) [144, 145]. Dose intense chemotherapy [105] or ASCT [146] elevates the risk of secondary leukemias, thus the incidence of secondary leukemia is higher in relapsed patients who require prolonged maintenance treatment with multiple courses of combination chemotherapy and transplantation.

**Table 5:** Secondary leukemia frequency, incidence, mortality rate in pediatric Hodgkin lymphoma

Patients (N)	Patients with SMN/ SMN no. (%)	Patients with leukemia (n)	% leukemia of total SMNs	Mean/ Median latency (leukemia) (yrs.)	Primary HL diagnosis period	CI of leukemia at 30 yrs. unless specified	SIR (Leukemia)	Mortality (leukemia) n (%)	Ref
2548	147 (5.8)	7 (5 AML, 1 ALL, 1 CML)	4.76	5.3	1978-2002	1.5*	10.4	6 (85%)	[145]
110	18 (16.3)	4 (3 AML, 1 ALL)	22.2	6.9	1970-90	4 (15 yrs plateau)	90.9	4 (100%)	[103]
930	102 (11)	9 (7 AML, 2 CML)	8.82	6.4	1960-90	-	21.49	9 (100%)	[154]
1380	73 (5.3)	27	36.9	4.3	1955-86	2.1	174.8	25 (93%)	[139]
2739	195 (7.1)	26	13.3	6.6	1935-94	-	33.3	-	[140]
182	28 (15.3)	2 (1 AML, 1 erythroleukemia)	7.1	10.68	1960-89	1.98	21.96	-	[155]
694	59 (8.5)	8 (7 AML)	13.55	4.3	1960-95	-	-	8 (100%)	[156]
1380	79 (5.7)	26 (24 AML, 1 ALL, 1 CML)	32.9	4.4	1955-86	2.8 (15 yrs plateau)	99.6	25 (96%)	[157]
1641	62 (3.8)	7	11.29	-	1943-87	0.8	17	-	[158]

ALL, Acute Lymphocytic Leukemia; AML, Acute Myeloid Leukemia; CI, Cumulative Incidence; CML, Chronic Myeloid Leukemia; SIR, Standardized Incidence Ratio; SMN, Second Malignant Neoplasm

* Cumulative incidence for leukemia and NHL together

The prognosis of secondary leukemia is significantly poorer compared to other hematological malignancies with a median survival of 2.5 to 4.5 months after diagnosis, and mortality rates that often reach 100% (Table 5). The OS and EFS at 3 years, specifically for therapy-related myelodysplastic syndrome/acute myeloid leukemia (tMDS/AML) secondary to primary cancers, are about 50% lower than that for *de novo* AML/MDS [147]. An increased risk of tMDS/AML is associated with mutations in genes that encode detoxifying enzymes (e.g., GSTM1, GSST1, and GSTP1), drug metabolizing enzymes (cP450), and proteins involved in DNA repair and apoptosis (XRCC1, p53) [91] (see section on genetic polymorphisms). In addition to genetic factors and chemotherapeutic agents, other unknown factors are likely involved in the development of secondary AML, as suggested by the occurrence of leukemias in seven HL patients who had not received mechlorethamine or etoposide [145]. In this regard, FGFR1 overexpression has been observed in lymph node biopsies of HL patients who developed AML [148]. Thus, assessment of FGFR as a potential molecular marker in pediatric HL patients who develop leukemia would be of value. Moreover, FGF2 can be overexpressed in adult HL patients [81], which highlights the importance of this signaling pathway, particularly in high-risk patients who are more likely to develop secondary AML.

Although recent successes in treating HL have been nothing short of revolutionary, we should not overlook the incidence of SMNs. Importantly, clinicians should comprehensively evaluate risks and benefits of available therapeutic approaches, and patients must be educated about the trade-offs between different treatment regimens and the importance of follow-up. Additionally, less aggressive regimens with low oncogenic potential that involve targeted therapies and lower doses of radiation should be given serious consideration when selecting HL treatment regimens. Genetic screening for potential SNPs that are implicated in HL may also offer progress toward personalized treatments for HL.

## CONCLUDING REMARKS

Most pediatric HL patients are cured with current first line risk-adapted and response-based chemotherapy. This high cure rate represents a success in the treatment of childhood cancer. Despite the high overall cure rates for HL patients, patients with early relapse or inadequate response are unlikely to achieve long-term remission with standard salvage therapy, and their prognosis remains grim. Therefore, identification of novel therapeutic targets to improve outcomes in this group remains an important challenge. The primary long-term complication of HL treatment is the development of secondary malignancy, which remains a cause for concern, especially for pediatric HL patients. As a means to avoid future side effects, recent clinical trials examined the effect of omitting RT in early stage patients. Although therapeutic sequelae and genetic factors are important in the development of secondary malignancies, other factors are involved, and, therefore, further studies are needed to identify biomarkers that indicate high-risk patients who will likely experience poor outcomes and are predisposed to develop secondary malignancies. We also need to understand the underlying mechanisms that are involved in the pathophysiology of high-risk HL and incorporate novel agents that can be personalized to achieve an initial cure and minimize late effects of HL therapy.
